# Disease-Modifying Therapies for Transthyretin Amyloid Cardiomyopathy: Current Evidence and Emerging Strategies

**DOI:** 10.14740/jocmr6603

**Published:** 2026-07-31

**Authors:** Meet Popatbhai Kachhadia, Amrit Gautam, Sarah Meadows, Smit R. Kotadiya, Roshani Aryal, Hasan Ilyas, Juber D. Shaikh, Gurnoor Gill, Samarth Shah, Jay Patel, Harshal A. Sanghvi

**Affiliations:** a Department of Neurology, Charles E. Schmidt College of Medicine, Florida Atlantic University, Boca Raton, FL, USA; b Internal Medicine Residency, Florida Atlantic University Charles E. Schmidt College of Medicine, Boca Raton, FL, USA; c Department of Medicine, G.M.E.R.S. Medical College, Junagadh, Gujarat, India; d Department of Internal Medicine, Kathmandu Medical College, Kathmandu, Nepal; e Department of Neurology, Prisma Health/University of South Carolina, Columbia, SC, USA; f Department of Technology and Clinical Trials, Advanced Research LLC, Pompano Beach, FL, USA; g Department of Biomedical Engineering, Florida Atlantic University, Boca Raton, FL, USA; h Department of Information Technology and Operations Management (ITOM), College of Business, Florida Atlantic University, Boca Raton, FL, USA

**Keywords:** Transthyretin amyloid cardiomyopathy, Disease-modifying therapy, Tafamidis, Ggene silencing, CRISPR

## Abstract

Transthyretin amyloid cardiomyopathy (ATTR-CM) is a progressive and life-threatening condition caused by extracellular deposition of misfolded transthyretin protein in cardiac tissue. Once considered rare and universally fatal, ATTR-CM has emerged as an increasingly recognized cause of heart failure, particularly among older adults. Recent therapeutic advances have transformed the management landscape from purely symptomatic care to disease-modifying interventions targeting multiple pathogenic mechanisms. This narrative review synthesizes current evidence on pharmacologic strategies for ATTR-CM, including transthyretin stabilizers such as tafamidis and acoramidis, gene-silencing therapies including patisiran, vutrisiran, and eplontersen, fibril disruptors, and emerging amyloid-depleting monoclonal antibodies and gene-editing approaches. We examine pivotal trial data demonstrating improvements in survival, functional capacity, and quality of life, alongside ongoing challenges related to safety, cost, and equitable access. The convergence of early diagnosis through improved imaging and biomarker strategies with targeted therapeutics has substantially changed the outlook for patients with a previously untreatable disease. Future directions emphasize combination therapies, biomarker-guided treatment selection, and precision medicine approaches tailored to disease genotype and phenotype.

## Introduction

Transthyretin amyloid cardiomyopathy (ATTR-CM) represents a paradigm shift in our understanding of restrictive heart disease. Historically dismissed as a rare genetic disorder confined to endemic regions, ATTR-CM is now recognized as a significant contributor to heart failure syndromes across diverse populations. The condition arises from progressive myocardial infiltration by amyloid fibrils composed of misfolded transthyretin protein, leading to ventricular wall thickening, diastolic dysfunction, conduction abnormalities, and ultimately heart failure and death [1]. ATTR-CM must be distinguished from light-chain (AL) amyloidosis, a plasma-cell dyscrasia with different treatment and prognosis; together these two types account for most cardiac amyloidosis. A validated noninvasive algorithm permits the diagnosis without endomyocardial biopsy. Grade 2 or 3 myocardial uptake on bone scintigraphy (technetium-labeled pyrophosphate, 3,3-diphosphono-1,2-propanodicarboxylic acid (DPD), or hydroxymethylene diphosphonate (HMDP)), combined with the absence of a monoclonal protein on serum and urine immunofixation and serum free light-chain assay, has a positive predictive value approaching 100% for ATTR-CM. In contrast, the presence of any monoclonal protein requires tissue typing to exclude AL disease [2].

Two distinct forms of ATTR-CM exist: wild-type ATTR-CM (ATTRwt-CM) and hereditary ATTR-CM (ATTRv-CM). ATTRwt-CM, historically referred to as senile cardiac amyloidosis, affecting predominantly elderly men. However, this longstanding characterization reflects historical case ascertainment rather than fixed biology; women are increasingly recognized as underdiagnosed, in part because wild-type disease in women tends to present with thinner left ventricular walls that fall below conventional hypertrophy thresholds, reducing the sensitivity of wall-thickness-based diagnostic criteria in women [3]. ATTRv-CM is caused by pathogenic variants in the transthyretin gene with variable age of onset and clinical phenotypes. Wild-type disease accounts for the majority of cases, with prevalence estimates ranging from 36.6% to 94.6% among diagnosed ATTR-CM cohorts [4]. The true burden of disease remains uncertain due to longstanding underdiagnosis, though recent studies suggest prevalence rates between 3.3% and 21% among patients with heart failure and preserved ejection fraction with left ventricular hypertrophy [5]. Recognized prevalence in the United States has risen substantially over the past decade as bone scintigraphy and disease awareness have expanded, a trend that reflects improved ascertainment rather than a true change in underlying incidence [6].

The molecular basis of ATTR amyloidosis centers on transthyretin, a homotetrameric protein synthesized primarily in the liver that normally functions as a carrier for thyroxine and retinol-binding protein. Destabilization of the native tetrameric structure leads to dissociation into monomers, which subsequently misfold and aggregate into amyloid fibrils. This rate-limiting dissociation step represents a critical therapeutic target [7]. In hereditary forms, amino acid substitutions destabilize the tetramer, accelerating amyloidogenesis, whereas in wild-type disease, age-related factors contribute to tetramer instability despite normal protein sequence.

Until recently, treatment options for ATTR-CM were limited to supportive heart failure management, with orthotopic heart transplantation and combined heart-liver transplantation reserved for highly selected patients. The United States Food and Drug Administration approved tafamidis for ATTR-CM in 2019, establishing that stabilizing the transthyretin tetramer could modify disease trajectory and improve clinical outcomes. The European Medicines Agency and Japan’s Pharmaceuticals and Medical Devices Agency subsequently approved tafamidis for this indication, with differences across regions in timing, approved formulation, and dosing. This advance catalyzed development of complementary therapeutic strategies targeting different points in the amyloidogenic cascade: gene silencing to reduce substrate production, fibril disruption to prevent aggregate formation, and amyloid depletion to remove existing deposits [5, 7].

The evolving therapeutic landscape for ATTR-CM now encompasses multiple mechanistic classes at various stages of clinical development. This review critically examines the evidence supporting current and emerging disease-modifying therapies, with emphasis on randomized controlled trial data, real-world effectiveness, safety considerations, and unmet clinical needs. We explore how rational combinations of these agents might optimize outcomes and discuss the role of precision diagnostics in guiding treatment selection [7]. The clinical phenotype at diagnosis is also shifting. As awareness and noninvasive diagnosis have improved, patients are increasingly identified at earlier disease stages, and recent pivotal trials have tended to enroll higher proportions of patients with New York Heart Association (NYHA) class I to II symptoms than the earliest tafamidis study, a change that affects the expected magnitude and time course of treatment benefit [8, 9].

## Methods

We conducted a narrative review of the medical literature focusing on disease-modifying pharmacologic therapies for ATTR-CM. PubMed was searched through December 2025 using the following terms: “transthyretin amyloid cardiomyopathy,” “ATTR cardiomyopathy,” “cardiac amyloidosis,” “tafamidis,” “acoramidis,” “patisiran,” “vutrisiran,” “inotersen,” “eplontersen,” “doxycycline,” “monoclonal antibody,” “CRISPR,” and “gene editing.” We prioritized phase 2 and phase 3 randomized controlled trials, large observational cohorts, mechanistic studies, and high-impact review articles. Inclusion criteria required human studies published in English-language peer-reviewed journals. Phase 1 studies were included when they represented first-in-human data for novel therapeutic classes. Case reports and conference abstracts were generally excluded unless they provided unique mechanistic insights unavailable in full publications. The search was conducted in PubMed/MEDLINE and supplemented by hand-searching the reference lists of identified articles and relevant society guidelines, and by querying ClinicalTrials.gov for the status of ongoing and completed trials (date range: database inception through December 2025). Titles and abstracts were screened against the inclusion and exclusion criteria stated above, followed by full-text review of potentially eligible records. Consistent with a narrative rather than a systematic review, we did not register a protocol, perform a multi-database systematic search with duplicate independent screening, or maintain a Preferred Reporting Items for Systematic Reviews and Meta-Analyses (PRISMA) flow diagram with a formal count of records identified and excluded. The narrative format was chosen because the objective is a mechanistic and clinical synthesis spanning heterogeneous designs (phase 1 through phase 3 trials, observational cohorts, and mechanistic studies), for which a single quantitative pooled estimate would be inappropriate. We recognize that narrative selection is susceptible to bias; to reduce this we gave precedence to randomized controlled trials and prespecified analyses and deliberately included negative, terminated, and inconclusive studies alongside positive trials.

## Pathophysiologic Basis for Therapeutic Targeting

**Figure 1:**
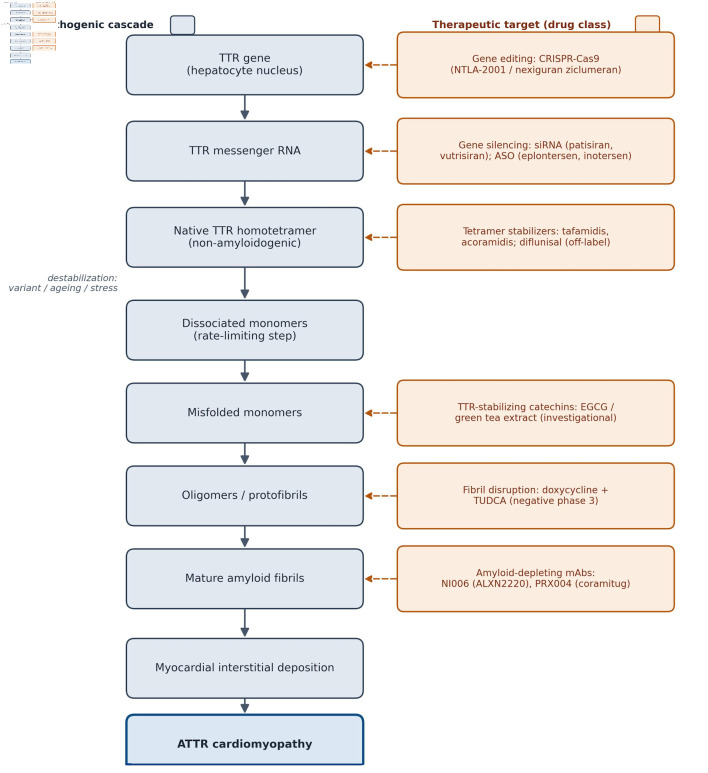
Amyloidogenic cascade in transthyretin amyloid cardiomyopathy and the points targeted by each therapeutic class. TTR: transthyretin; siRNA: small interfering RNA; ASO: antisense oligonucleotide; CRISPR: clustered regularly interspaced short palindromic repeats; TUDCA: tauroursodeoxycholic acid.

Understanding the molecular pathogenesis of transthyretin amyloidosis illuminates the rationale for distinct therapeutic approaches. Native transthyretin exists as a homotetramer formed by noncovalent association of four identical monomers, each comprising 127 amino acids. The quaternary structure features a central hydrophobic channel capable of binding two thyroxine molecules. Tetramer stability is the key determinant of amyloidogenic potential, with destabilization triggering dissociation into monomers that undergo conformational changes and aggregate into oligomers, protofibrils, and ultimately mature amyloid fibrils [10] (Fig. 1).

The kinetic barrier to tetramer dissociation is naturally high for wild-type transthyretin at physiological pH, explaining why ATTRwt-CM manifests predominantly in advanced age when age-related factors reduce tetramer stability. More than 150 pathogenic variants have been identified, many of which significantly destabilize the tetramer, lowering the activation energy for dissociation and leading to earlier disease onset. Interestingly, the V122I variant, which is present in approximately 3.4% of individuals of African ancestry in the United States, exhibits only modest destabilization yet confers substantially increased lifetime risk of ATTR-CM [11].

Once deposited in the myocardial interstitium, amyloid fibrils exert both direct mechanical effects through tissue expansion and increased stiffness and indirect toxic effects through cellular stress responses, oxidative injury, and disruption of normal extracellular matrix architecture. The cumulative burden of amyloid correlates with disease severity and prognosis, though the contribution of potentially toxic oligomeric intermediates remains an area of active investigation [10, 11].

## Transthyretin Stabilizers

### Tafamidis

Tafamidis represents the first and currently only regulatory-approved therapy definitively shown to reduce mortality and cardiovascular hospitalizations in ATTR-CM. The drug functions by binding selectively to the thyroxine-binding sites within the transthyretin tetramer, kinetically stabilizing the quaternary structure and dramatically reducing the rate of tetramer dissociation. By maintaining tetramers in their native, non-amyloidogenic conformation, tafamidis halts the rate-limiting step in fibril formation. The pivotal ATTR-ACT trial, a randomized double-blind placebo-controlled study, enrolled 441 patients with ATTRwt-CM or ATTRv-CM and NYHA functional class I to III symptoms across 13 countries. Participants were randomized 2:1:2 to tafamidis meglumine 80 mg, tafamidis meglumine 20 mg, or placebo for 30 months. The primary endpoint combined all-cause mortality and cardiovascular-related hospitalizations. Results demonstrated that tafamidis significantly reduced all-cause mortality compared with placebo (29.5% vs 42.9%; hazard ratio 0.70, 95% confidence interval (CI), 0.51 to 0.96). Tafamidis also reduced the frequency of cardiovascular hospitalizations (0.48 vs 0.70 per year; relative risk ratio 0.68, 95% CI, 0.56 to 0.81). Importantly, the benefits were most pronounced when therapy was initiated early in the disease course, underscoring the preventive rather than reparative nature of tetramer stabilization. Secondary endpoints revealed preservation of functional capacity measured by 6-min walk distance and maintenance of quality of life assessed by the Kansas City Cardiomyopathy Questionnaire, both of which declined in the placebo group [12, 13].

Long-term extension data from patients transitioning from ATTR-ACT into an open-label phase provide compelling evidence for early treatment initiation. Patients who received continuous tafamidis from trial enrollment demonstrated superior survival compared with those initially randomized to placebo who later crossed over to active treatment. Median survival was not reached in the continuous tafamidis group versus 35.8 months in the delayed treatment group, with a median follow-up exceeding 50 months. Among patients with NYHA class III symptoms at baseline, a population in which the benefit of disease-modifying therapy had been questioned, continuous tafamidis treatment reduced mortality risk by 36% compared with delayed treatment (hazard ratio 0.64, 95% CI, 0.41 to 0.99) over approximately 5 years of follow-up. Tafamidis is available in two bioequivalent formulations: tafamidis meglumine 80 mg (Vyndaqel) and tafamidis free acid 61 mg (Vyndamax), administered orally once daily. The drug is generally well tolerated, with adverse events comparable to placebo in controlled trials. No routine laboratory monitoring beyond standard heart failure management is required [14, 15].

Table 1 [12, 14, 15] summarizes the major clinical trials of tafamidis in ATTR-CM, demonstrating mortality benefit and long-term survival with this first-in-class transthyretin stabilizer.

**Table 1:** Major Clinical Trials of Tafamidis in ATTR-CM

Study	Trial name	Design	Sample size	Duration	Primary endpoint	Key outcome
Maurer et al, 2018 [12]	ATTR-ACT	Phase 3 RCT	441	30 months	Composite of all-cause mortality and cardiovascular hospitalizations	Tafamidis reduced all-cause mortality (HR 0.70; 95% CI, 0.51–0.96) and decreased the annual rate of cardiovascular hospitalizations (0.48 vs 0.70 per year; RR 0.68, 95% CI, 0.56–0.81) compared with placebo
Damy et al, 2020 [14]	ATTR-ACT open-label extension	Open-label extension	400+	50+ months	Long-term survival	Continuous tafamidis from baseline was associated with longer median survival; median survival was not reached in the continuous group versus 35.8 months with delayed initiation
Elliott et al, 2023 [15]	NYHA III *post-hoc* analysis of ATTR-ACT	Post-hoc analysis	142	About 5 years	All-cause mortality	In patients with NYHA class III symptoms at baseline, continuous tafamidis reduced mortality risk by 36% compared with delayed treatment (HR 0.64; 95% CI, 0.41–0.99)

Cardiovascular hospitalizations (CV hosp) refer to hospital admissions for cardiovascular causes, including worsening heart failure, arrhythmias, acute coronary syndromes, or other cardiac events requiring hospitalization. ATTR-CM: transthyretin amyloid cardiomyopathy; RCT: randomized controlled trial; HR: hazard ratio; CI: confidence interval; RR: relative risk; NYHA: New York Heart Association.

### Acoramidis

Acoramidis (AG10) is a next-generation selective transthyretin stabilizer designed to mimic the protective T119M variant, one of the rare naturally occurring transthyretin mutations that enhances tetramer stability and protects against amyloidosis. Preclinical studies demonstrated that acoramidis achieves near-complete stabilization of transthyretin tetramers, potentially offering greater potency than tafamidis [8, 16].

The phase 3 ATTRibute-CM trial evaluated acoramidis 800 mg twice daily versus placebo in 632 patients with ATTR-CM over 30 months. The primary analysis employed a hierarchical composite endpoint integrating all-cause mortality, cardiovascular-related hospitalizations, N-terminal pro-B-type natriuretic peptide (NT-proBNP) change, and 6-min walk distance. Acoramidis demonstrated statistically significant and clinically meaningful superiority on the primary endpoint (win ratio 1.8; 95% CI, 1.4 to 2.2). All-cause mortality or first cardiovascular hospitalization occurred less frequently with acoramidis than placebo (hazard ratio 0.64; 95% CI, 0.50 to 0.83), mirroring the magnitude of benefit observed with tafamidis in ATTR-ACT [8].

Interim results from the open-label extension study revealed sustained benefits with longer treatment duration. At 42 months, the hazard ratio for all-cause mortality or cardiovascular hospitalization was 0.57 (95% CI, 0.46 to 0.72), reinforcing the importance of early intervention and continued therapy. Functional capacity measured by 6-min walk distance was preserved in the acoramidis group while declining in placebo, and quality of life scores similarly favored active treatment [16].

Safety analysis showed acoramidis to be well tolerated, with treatment-emergent adverse events balanced between groups. A phase 3 study conducted specifically in Japanese patients with ATTR-CM confirmed the favorable efficacy and safety profile observed in the global trial, with no deaths reported over the 30-month study period and improvements in functional and quality-of-life measures [17].

The similar clinical efficacy of acoramidis and tafamidis, despite potential differences in molecular stabilization potency, raises intriguing questions about the relationship between *in vitro* tetramer stabilization and clinical outcomes. Whether maximal stabilization confers incremental benefit over submaximal stabilization remains an area requiring direct comparative investigation [16, 17].

Table 2 [16, 18, 19] summarizes the pivotal clinical trials of acoramidis in ATTR-CM, a next-generation transthyretin stabilizer demonstrating efficacy through a hierarchical composite endpoint.

**Table 2:** Major Clinical Trials of Acoramidis in ATTR-CM

Study	Trial name	Design	Sample size	Duration	Primary endpoint	Key outcome
Gillmore et al. [18]	ATTRibute-CM	Phase 3 RCT	632	30 months	Hierarchical composite endpoint	Acoramidis improved the hierarchical composite endpoint (all-cause mortality, cardiovascular hospitalizations, NT-proBNP change, and 6-min walk distance) versus placebo (win ratio, 1.8; 95% CI, 1.4–2.2); all-cause mortality or first cardiovascular hospitalization occurred less frequently with acoramidis (HR, 0.64; 95% CI, 0.50–0.83)
Judge et al, 2025 [12]	ATTRibute-CM open-label extension	Open-label extension	600+	42 months	Composite of all-cause mortality and cardiovascular hospitalizations	Long-term treatment with acoramidis reduced the risk of all-cause mortality or first cardiovascular hospitalization (HR, 0.57; 95% CI, 0.46–0.72) and maintained functional capacity measured by 6-min walk distance and quality of life over 42 months
Endo et al, 2026 [19]	Japanese phase 3 acoramidis study	Phase 3 open-label	25	30 months	Safety and efficacy	In Japanese patients with ATTR-CM, acoramidis showed favorable safety with no deaths reported over the 30-month study period and improvements in functional status and quality-of-life measures

Cardiovascular hospitalizations (CV hosp) refer to hospital admissions for cardiovascular causes. RCT: randomized controlled trial; HR: hazard ratio; CI: confidence interval; NT-proBNP: N-terminal pro-B-type natriuretic peptide; ATTR-CM: transthyretin amyloid cardiomyopathy.

## Gene Silencing Therapies

An alternative strategy to stabilizing circulating transthyretin involves suppressing hepatic synthesis of the protein, thereby reducing substrate available for amyloid formation. Two complementary RNA-targeted approaches have been developed: small interfering RNA (siRNA) therapeutics and antisense oligonucleotides (ASOs), both of which induce degradation of transthyretin mRNA and achieve profound reductions in serum transthyretin levels [17, 20]. Because transthyretin is the principal serum carrier of retinol-binding protein, profound suppression of hepatic transthyretin lowers circulating retinol. Patients receiving gene silencers are therefore given vitamin A supplementation, commonly 2,500 to 3,000 IU daily, with monitoring for ocular symptoms of deficiency [21].

### Patisiran

Patisiran, a lipid nanoparticle-encapsulated siRNA, was the first RNA interference therapy approved for treatment of hereditary transthyretin amyloidosis with polyneuropathy. The phase 3 APOLLO trial in patients with ATTRv polyneuropathy demonstrated that patisiran not only stabilized neurologic function but also improved cardiac biomarkers in participants with baseline cardiac involvement [20]. These signals of cardiac benefit prompted dedicated investigation in ATTR-CM.

The APOLLO-B trial enrolled 360 patients with ATTR-CM (both wild-type and hereditary) randomized 1:1 to patisiran 0.3 mg/kg intravenously every 3 weeks or placebo for 12 months. The primary endpoint was change in 6-min walk distance. Although the trial did not meet the prespecified threshold for statistical significance on the primary endpoint using the original analysis plan, revised analyses accounting for mortality as a competing risk demonstrated a treatment benefit, with patisiran attenuating decline in 6-min walk distance by approximately 15 m compared with placebo [22].

Secondary endpoints provided consistent evidence of disease modification. Patisiran preserved health-related quality of life assessed by the Kansas City Cardiomyopathy Questionnaire Overall Summary score, whereas placebo-treated patients experienced clinically meaningful deterioration. Cardiac biomarkers including NT-proBNP and high-sensitivity troponin T showed favorable trends with patisiran. Echocardiographic parameters, particularly global longitudinal strain, suggested preservation of systolic function in the treatment group [23].

The safety profile of patisiran in APOLLO-B reflected experience from the polyneuropathy trials, with mild infusion-related reactions representing the most common adverse event. These reactions typically occurred during the first infusion and were manageable with premedication protocols.

### Vutrisiran

Vutrisiran is an enhanced stability siRNA conjugated to a triantennary N-acetylgalactosamine moiety that enables subcutaneous administration and quarterly dosing. The conjugate facilitates hepatocyte-specific uptake via the asialoglycoprotein receptor, eliminating the need for lipid nanoparticle formulation. In the HELIOS-A trial, which evaluated vutrisiran in patients with hereditary ATTR polyneuropathy, vutrisiran demonstrated non-inferior efficacy to patisiran with the advantage of subcutaneous delivery and less frequent dosing [24].

Building on these results, the phase 3 HELIOS-B trial assessed vutrisiran 25 mg subcutaneously every 3 months versus placebo in 664 patients with ATTR-CM over 30 months. The trial employed a hierarchical composite endpoint evaluating all-cause mortality, recurrent cardiovascular events, change in NT-proBNP, and change in 6-min walk distance. Vutrisiran demonstrated statistically significant superiority on the primary endpoint, with a win ratio of 1.7 (95% CI, 1.4 to 2.1). The risk of all-cause mortality or recurrent cardiovascular events was reduced by approximately 33% (hazard ratio = 0.67; 95% CI, 0.52 to 0.87) [9].

Echocardiographic substudy data revealed that vutrisiran attenuated increases in left ventricular wall thickness and left ventricular mass index compared with placebo over 30 months. Additionally, vutrisiran preserved measures of systolic function including left ventricular ejection fraction and global longitudinal strain, and reduced deterioration in diastolic function parameters [25]. These structural and functional benefits complement the clinical endpoint findings and suggest that reduction of circulating transthyretin levels may allow gradual regression of amyloid burden or at minimum prevent further accumulation.

The subcutaneous route of administration and quarterly dosing schedule offer substantial logistical advantages over intravenous therapies, particularly relevant for chronic lifelong treatment and highlighted during the coronavirus disease 2019 (COVID-19) pandemic when clinic access was challenging.

### Eplontersen

Eplontersen is a ligand-conjugated ASO that binds transthyretin mRNA, leading to RNase H-mediated degradation. Like vutrisiran, eplontersen is conjugated to N-acetylgalactosamine for hepatocyte targeting and is administered subcutaneously, though on a monthly schedule. The phase 3 NEURO-TTRansform trial evaluated eplontersen in 168 patients with hereditary ATTR polyneuropathy. Compared with an external placebo cohort from the prior inotersen trial, eplontersen significantly reduced progression of neuropathy measured by the modified Neuropathy Impairment Score plus 7 and improved quality of life. Exploratory cardiac analyses in participants with baseline cardiomyopathy showed reductions in NT-proBNP and stabilization of echocardiographic parameters including global longitudinal strain and left ventricular wall thickness [26].

These encouraging signals of cardiac benefit supported initiation of the CARDIO-TTRansform trial, an ongoing phase 3 placebo-controlled study evaluating eplontersen in patients with ATTR-CM. The trial is assessing hard clinical endpoints including all-cause mortality, cardiovascular hospitalizations, and functional capacity. In parallel, the EPIC-ATTR trial is evaluating eplontersen specifically in Chinese patients with ATTR-CM, with primary completion anticipated in 2025 and final data expected in 2027. Monthly subcutaneous dosing positions eplontersen between the 3-week intravenous schedule of patisiran and the quarterly subcutaneous schedule of vutrisiran, potentially balancing sustained transthyretin suppression with dosing convenience [27].

Table 3 [9, 22, 26] summarizes the gene silencing therapies for ATTR-CM, including siRNA and ASO agents that reduce hepatic transthyretin production.

**Table 3:** Gene silencing Therapies in ATTR-CM

Study	Trial name	Agent	Route	Dosing	Sample size	Duration	Key outcome
Maurer et al, 2023 [22]	APOLLO-B	Patisiran	IV	Every 3 weeks	360	12 months	Patisiran attenuated the decline in 6-min walk distance versus placebo (treatment effect about 15 m) and preserved health-related quality of life, with favorable trends in cardiac biomarkers including NT-proBNP and high-sensitivity troponin T
Fontana et al, 2025 [9]	HELIOS-B	Vutrisiran	SC	Every 3 months	664	30 months	Vutrisiran improved the hierarchical composite endpoint with win ratio of 1.7 (95% CI, 1.4–2.1), reducing the risk of all-cause death or recurrent cardiovascular events by approximately 33% (HR, 0.67; 95% CI, 0.52–0.87)
Coelho et al, 2023 [26]	NEURO-TTRansform	Eplontersen	SC	Monthly	168	18 months	Eplontersen slowed neuropathy progression; exploratory cardiac analyses showed reductions in NT-proBNP and stabilization of global longitudinal strain and left ventricular wall thickness

ATTR-CM: transthyretin amyloid cardiomyopathy; IV: intravenous; SC: subcutaneous; HR: hazard ratio; CI: confidence interval; NT-proBNP: N-terminal pro-B-type natriuretic peptide.

### Inotersen

Inotersen, an unconjugated ASO administered subcutaneously weekly, was approved for treatment of hereditary ATTR polyneuropathy based on the NEURO-TTR trial. Although the drug demonstrated efficacy in slowing neuropathy progression and improving quality of life, clinical development was complicated by serious adverse events including thrombocytopenia and glomerulonephritis requiring intensive monitoring protocols. Limited cardiac substudies suggested potential benefit on biomarkers, but the need for weekly platelet monitoring and renal function surveillance limits practical applicability in the ATTR-CM population, particularly given availability of alternative gene silencing agents with more favorable safety profiles [28].

### Historical context: revusiran

The ENDEAVOUR trial, which evaluated the siRNA agent revusiran in patients with hereditary ATTR-CM, was terminated early in 2016 due to an imbalance in mortality favoring placebo (hazard ratio 5.3; 95% CI, 1.2 to 22.8). Despite extensive investigation, no clear mechanism for the excess mortality was identified, and the deaths were not attributed to a specific drug-related toxicity. This setback temporarily cast doubt on the gene silencing strategy for ATTR-CM but ultimately spurred more rigorous trial design and safety monitoring protocols that benefited subsequent siRNA development programs. The success of patisiran and vutrisiran vindicated the therapeutic approach while highlighting the importance of careful formulation and comprehensive safety assessment [29].

## Fibril Disruption: Doxycycline Plus Tauroursodeoxycholic Acid

The combination of doxycycline, a tetracycline antibiotic, and tauroursodeoxycholic acid (TUDCA), a bile acid derivative, was investigated based on preclinical evidence suggesting that the combination could disrupt amyloid fibril formation and exert cytoprotective effects. A phase 2 open-label study in 20 patients with ATTR amyloidosis, predominantly wild-type, administered doxycycline 100 mg twice daily plus TUDCA 250 mg three times daily for 12 months. The regimen was generally well tolerated, and most patients demonstrated disease stabilization, raising hope for a readily available and inexpensive therapeutic option [30].

However, the subsequent phase 3 DOXY-TUDCA trial (NCT03481972), a randomized controlled study comparing standard supportive therapy alone versus standard therapy plus doxycycline-TUDCA in patients with ATTRwt-CM, failed to demonstrate a survival benefit with the addition of the drug combination. The trial concluded that doxycycline plus TUDCA does not provide significant clinical benefit in ATTR-CM and is not recommended as standard therapy [31].

The discrepancy between early positive signals and the negative definitive trial underscores the necessity of placebo-controlled trials in ATTR-CM, where disease progression can be variable and spontaneous stabilization is uncommon but possible. The doxycycline-TUDCA combination is not currently part of evidence-based management [30, 31].

## Investigational and Lower-Cost Approaches

Several lower-cost or repurposed interventions have been explored, although none is supported by randomized evidence in ATTR-CM and none is currently recommended as disease-modifying therapy. Epigallocatechin-3-gallate (EGCG), a green tea polyphenol that inhibits transthyretin fibril formation *in vitro*, was associated with stabilization of left ventricular mass on cardiac magnetic resonance in small observational studies of patients with ATTR-CM followed for 12 months [32, 33]. These reports were uncontrolled, enrolled fewer than 20 patients each, and relied on surrogate imaging endpoints, so they cannot establish clinical benefit.

Low-dose cardiac radiotherapy has been proposed as a means of reducing myocardial amyloid through effects on the local immune and inflammatory response. A prospective first-in-human series of five patients with ATTRwt-CM (mean age 87 years) treated with 10 Gy in five fractions reported a directional decrease in cardiac amyloid signal on positron emission tomography (PET) imaging, with no efficacy conclusions drawn given the sample size [34]. This is early hypothesis-generating data only.

Diflunisal, a nonsteroidal anti-inflammatory drug (NSAID) that stabilizes the transthyretin tetramer, slowed neurologic progression in a randomized trial in hereditary ATTR polyneuropathy (difference in the modified Neuropathy Impairment Score plus 7 of 16.3 points versus placebo at 2 years, P < 0.001) [35]. Evidence in ATTR-CM is limited to small and observational reports, and use is constrained by the renal, gastrointestinal, and fluid-retention risks of chronic NSAID exposure in an older heart failure population. Diflunisal is sometimes used off-label where approved stabilizers are unavailable, but it is not approved for ATTR-CM.

## Amyloid-Depleting Therapies

The agents discussed thus far primarily prevent new amyloid formation by stabilizing transthyretin or reducing its production. A fundamentally different approach involves actively clearing existing amyloid deposits from tissues, potentially reversing organ damage rather than merely halting progression. Two strategies are under investigation: monoclonal antibodies targeting amyloid fibrils and gene editing to permanently silence hepatic transthyretin production.

### Monoclonal antibodies

NI006 (ALXN2220) is a recombinant human monoclonal antibody designed to bind specifically to misfolded and aggregated transthyretin while sparing the native tetrameric protein. Upon binding, the antibody-amyloid complex is recognized and cleared by phagocytic immune cells, gradually depleting tissue amyloid burden. A phase 1 proof-of-concept study evaluated NI006 in 41 patients with ATTR-CM, administering ascending doses up to 60 mg/kg intravenously monthly for 12 months. The drug was well tolerated without dose-limiting toxicity. Cardiac imaging using both scintigraphy and cardiac magnetic resonance demonstrated substantial reductions in myocardial amyloid signal at doses of 10 mg/kg or higher. Cardiac biomarkers including NT-proBNP and high-sensitivity troponin T declined from baseline, and no drug-related serious adverse events occurred. These encouraging results represent the first clinical evidence that removal of deposited amyloid is achievable and potentially beneficial [36].

Phase 2 and phase 3 trials are currently evaluating NI006 in larger cohorts to determine whether amyloid depletion translates into improvements in hard clinical endpoints including mortality and hospitalizations. If successful, antibody-mediated amyloid clearance could complement transthyretin stabilizers or gene silencers, addressing both substrate reduction and deposit removal. PRX004 (coramitug, NNC6019-0001) represents another investigational monoclonal antibody targeting misfolded transthyretin. Phase 1 data in patients with hereditary ATTR amyloidosis showed the antibody to be well tolerated with dose-proportional pharmacokinetics. Exploratory efficacy analyses suggested stabilization or improvement in global longitudinal strain and neuropathy impairment scores over 9 months. A phase 2 randomized controlled trial in ATTR-CM is ongoing to rigorously assess clinical efficacy (NCT05442047) [36, 37].

Table 4 [36, 37] summarizes the monoclonal antibody approaches for ATTR amyloidosis, which target misfolded or aggregated transthyretin to promote clearance of existing amyloid deposits.

**Table 4:** Monoclonal Antibody Trials in ATTR Amyloidosis

Study	Trial name	Antibody	Target	Phase	Sample size	Key finding	Status
Garcia-Pavia et al, 2023 [36]	NI006 phase 1	NI006 (ALXN2220)	Aggregated TTR	1	41	Monthly NI006 infusions (up to 60 mg/kg) were well tolerated. At doses ≥ 10 mg/kg, cardiac imaging demonstrated substantial reductions in myocardial amyloid burden with decreases in NT-proBNP and high-sensitivity troponin T	Phase 2/3 ongoing
Richards et al, 2024 [37]	PRX004 phase 1 dose-escalation	PRX004 (coramitug, NNC6019-0001)	Misfolded TTR	1	26	PRX004 was well tolerated with dose-proportional pharmacokinetics. Exploratory analyses suggested stabilization or improvement in global longitudinal strain and neuropathy impairment scores over 9 months	Phase 2 ongoing (NCT05442047)

TTR: transthyretin; MRI: magnetic resonance imaging; NT-proBNP: N-terminal pro-B-type natriuretic peptide; GLS: global longitudinal strain.

### Gene editing with clustered regularly interspaced short palindromic repeats (CRISPR)-Cas9

NTLA-2001 (nexiguran ziclumeran, nex-z) represents a fundamentally different approach: single-dose *in vivo* gene editing using CRISPR-Cas9 technology to permanently inactivate the transthyretin gene. The therapeutic agent comprises lipid nanoparticles encapsulating messenger RNA encoding Cas9 endonuclease and a single-guide RNA targeting the *TTR* gene. Following intravenous infusion, the nanoparticles are preferentially taken up by hepatocytes, where the CRISPR machinery edits the chromosomal *TTR* gene, creating insertions or deletions that disrupt gene function. The first-in-human phase 1 trial enrolled patients with hereditary ATTR amyloidosis with polyneuropathy. A single infusion of NTLA-2001 at doses of 0.3 mg/kg or higher resulted in rapid, profound, and durable reductions in serum transthyretin levels. At the highest dose tested, mean serum transthyretin reductions exceeded 90% and remained stable through 12 months of follow-up. The therapy was generally well tolerated, with mild to moderate infusion-related reactions representing the most common adverse events [38].

A parallel phase 1 study evaluated nexiguran ziclumeran in patients with ATTR-CM, demonstrating similarly robust and sustained transthyretin reductions. Beyond biochemical efficacy, preliminary data suggested improvements or stabilization in functional capacity, quality of life, and cardiac biomarkers, though definitive efficacy determination awaits completion of ongoing trials. An extension study tracking participants for 24 months post-infusion has confirmed durability of transthyretin knockdown without loss of effect, supporting the potential for one-time curative therapy. Permanent disease modification with a single treatment is an attractive prospect, though several questions remain. Long-term safety of chromosomal editing, potential off-target effects, and the clinical consequences of near-total transthyretin depletion require ongoing surveillance. Additionally, while hereditary forms of ATTR-CM may be particularly well-suited to gene editing, applicability to wild-type disease, which comprises the majority of cases, is less straightforward given that the underlying issue is not genetic mutation but age-related protein destabilization. Regulatory agencies have placed clinical holds on some CRISPR programs following isolated adverse events, highlighting the need for cautious development and rigorous monitoring. Nonetheless, the technology represents a potential paradigm shift from chronic pharmacotherapy to one-time intervention [38, 39].

Table 5 [38, 39] summarizes the CRISPR-Cas9 gene editing trials for ATTR amyloidosis, demonstrating durable transthyretin reduction with single-dose administration.

**Table 5:** CRISPR-Cas9 Gene Editing Trials

Study	Trial name	Population	Sample size	Dose	TTR reduction	Duration	Key outcome	Safety
Gillmore et al, 2021 [38]	NTLA-2001 phase 1 ATTRv-PN	Hereditary ATTR with polyneuropathy	36	0.3–1.0 mg/kg	Up to 93%	12 months	Single-dose NTLA-2001 achieved up to about 93% sustained reduction in serum TTR at 12 months	Well tolerated; mild to moderate infusion-related reactions were the most common adverse events
Fontana et al, 2024 [39]	Nexiguran ziclumeran phase 1 ATTR-CM	ATTR cardiomyopathy	36	0.7–1.0 mg/kg	Up to 90%	24 months	Single-dose nex-z produced up to about 90% sustained TTR reduction over 24 months with improvements or stabilization in functional capacity and cardiac biomarkers	Mild infusion reactions; well tolerated with no drug-related serious adverse events

CRISPR: clustered regularly interspaced short palindromic repeats; TTR: transthyretin; ATTRv-PN: hereditary ATTR with polyneuropathy; ATTR-CM: transthyretin amyloid cardiomyopathy; NT-proBNP: N-terminal pro-B-type natriuretic peptide; GLS: global longitudinal strain.

## Comparative Perspectives and Treatment Selection

**Figure 2:**
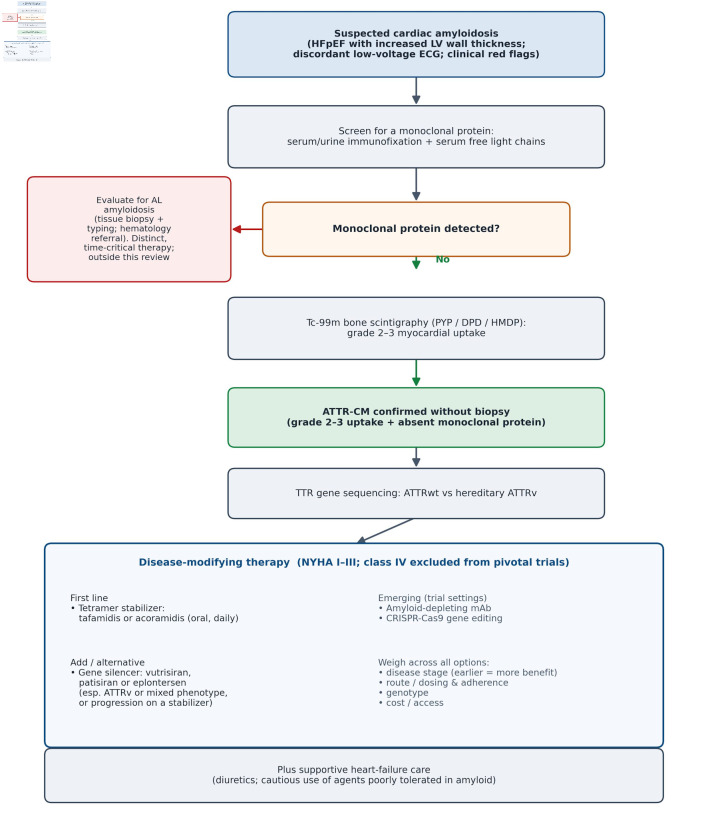
Diagnostic and treatment-selection algorithm for transthyretin amyloid cardiomyopathy. ATTR-CM: transthyretin amyloid cardiomyopathy; NYHA: New York Heart Association; LV: left ventricular; HFpEF: heart failure with preserved ejection fraction; PYP: technetium-99m pyrophosphate; DPD: 3,3-diphosphono-1,2-propanodicarboxylic acid; HMDP: hydroxymethylene diphosphonate; ECG: electrocardiogram.

The expanding therapeutic armamentarium for ATTR-CM raises the question of how to select among available and emerging options (Fig. 2). Tafamidis currently holds the strongest evidence base with demonstrated mortality reduction and regulatory approval, making it the standard of care for eligible patients. Acoramidis offers a closely comparable clinical profile, and its availability provides an alternative for patients who cannot access tafamidis.

**Figure 3:**
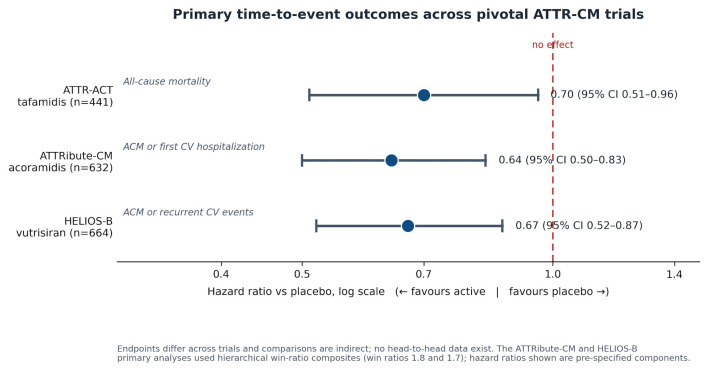
Primary or key composite hazard ratios from the pivotal disease-modifying therapy trials in ATTR-CM. Endpoints, follow-up durations, and analytic methods differ across trials and no head-to-head comparisons exist, so the estimates are not directly comparable. Created by the authors. CI: confidence interval; ATTR-CM: transthyretin amyloid cardiomyopathy; CV: cardiovascular.

Direct comparison across agents is limited by differences in trial design and endpoint definition, so apparent similarities in effect size should be interpreted cautiously. ATTR-ACT used a finite combination of all-cause mortality and cardiovascular hospitalization and reported an all-cause mortality hazard ratio of 0.70 (95% CI, 0.51 to 0.96). ATTRibute-CM used a hierarchical (win-ratio) primary analysis combining death, cardiovascular hospitalization, and changes in NT-proBNP and 6-min walk distance, with a hazard ratio for all-cause mortality or first cardiovascular hospitalization of 0.64 (95% CI, 0.50 to 0.83). HELIOS-B used a hierarchical composite and reported a hazard ratio for all-cause mortality or recurrent cardiovascular events of 0.67 (95% CI, 0.52 to 0.87). Because the endpoints, follow-up durations, and analytic methods differ, and because no head-to-head trials exist, these estimates cannot be equated: the apparent convergence is hypothesis-generating rather than evidence of equivalence (Fig. 3). Table 6 grades the disease-modifying and investigational therapies for ATTR-CM by development phase, regulatory status, primary endpoint type, and demonstrated clinical benefit.

**Table 6:** Evidence Grading of Disease-Modifying and Investigational Therapies for ATTR-CM

Agent	Mechanism class	Highest phase in ATTR-CM	Regulatory status (ATTR-CM)	Primary endpoint type	Mortality or CV-hospitalization benefit demonstrated
Tafamidis	Tetramer stabilizer	Phase 3 (ATTR-ACT)	Approved (FDA, EMA, PMDA)	Finite composite of all-cause mortality and CV hospitalization	Yes; all-cause mortality HR 0.70 (0.51–0.96)
Acoramidis	Tetramer stabilizer	Phase 3 (ATTRibute-CM)	Approved (FDA 2024; other regions vary)	Hierarchical win-ratio composite	CV outcome benefit; all-cause mortality alone not independently significant
Patisiran	siRNA gene silencer	Phase 3 (APOLLO-B)	Approved for ATTRv-PN; not approved for ATTR-CM	6-min walk distance (continuous)	No mortality benefit shown in ATTR-CM
Vutrisiran	siRNA gene silencer	Phase 3 (HELIOS-B)	Approved for ATTR-CM (FDA 2025)	Hierarchical composite	Yes; all-cause mortality or recurrent CV events HR 0.67 (0.52–0.87)
Eplontersen	Antisense oligonucleotide gene silencer	Phase 3 ongoing (CARDIO-TTRansform)	Approved for ATTRv-PN; ATTR-CM under study	Hard clinical endpoints (pending)	Pending
Inotersen	Antisense oligonucleotide gene silencer	Phase 3 in ATTRv-PN (NEURO-TTR)	Approved for ATTRv-PN; not for ATTR-CM	Neuropathy endpoints (not cardiac)	Not established in ATTR-CM; use limited by safety
Doxycycline plus TUDCA	Fibril disruption	Phase 3 (DOXY-TUDCA)	Not approved	Survival	No; negative phase 3 trial
NI006 (ALXN2220)	Anti-amyloid monoclonal antibody	Phase 1 complete; phase 3 ongoing	Investigational	Imaging and biomarker (phase 1)	Not yet; outcome trials ongoing
PRX004 (coramitug)	Anti-amyloid monoclonal antibody	Phase 1 complete; phase 2 ongoing	Investigational	Exploratory (GLS, biomarkers)	Not established
NTLA-2001/nexiguran ziclumeran	CRISPR-Cas9 gene editing	Phase 1 complete; phase 3 ongoing	Investigational	TTR knockdown; clinical endpoints pending	Not yet established
Diflunisal	Tetramer stabilizer (NSAID)	RCT in ATTRv-PN; observational in ATTR-CM	Not approved for ATTR-CM (used off-label)	Neuropathy endpoint (PN trial)	No cardiac RCT; not established
EGCG (green tea)	Fibril-formation inhibitor (polyphenol)	Small observational studies only	Not a recognized drug indication	Imaging surrogate	No; uncontrolled data only

Status reflects ATTR-CM unless otherwise noted and varies by jurisdiction and date. HR: hazard ratio; CV: cardiovascular; ATTRv-PN: hereditary ATTR with polyneuropathy; GLS: global longitudinal strain; TUDCA: tauroursodeoxycholic acid; NSAID: nonsteroidal anti-inflammatory drug; EGCG: epigallocatechin-3-gallate; FDA: Food and Drug Administration; EMA: European Medicines Agency; PMDA: Pharmaceuticals and Medical Devices Agency; RCT: randomized controlled trial; siRNA: small interfering RNA; CRISPR: clustered regularly interspaced short palindromic repeats; ATTR-CM: transthyretin amyloid cardiomyopathy.

Table 7 [7–9, 12, 22, 26, 30, 36–39] summarizes the distinct mechanistic classes of disease-modifying therapies for ATTR-CM, highlighting their respective therapeutic targets and key representative agents.

**Table 7:** Therapeutic Strategies for Transthyretin Amyloid Cardiomyopathy

Treatment category	Drugs	Mechanism of action
TTR stabilization	Tafamidis, acoramidis	Stabilize TTR tetramer by binding to thyroxine-binding sites, preventing tetramer dissociation and reducing the rate-limiting step in fibril formation [7, 8, 12]
Gene silencing	Patisiran, vutrisiran, eplontersen	Reduce hepatic TTR synthesis through RNA interference (siRNA) or antisense oligonucleotide mechanisms, inducing degradation of TTR mRNA and achieving profound reductions in serum TTR levels [9, 22, 26]
Fibril disruption	Doxycycline-TUDCA	Disrupt amyloid fibril formation and exert cytoprotective effects (not currently recommended as evidence-based therapy following negative phase 3 trial results) [30]
Amyloid depletion	NI006, PRX004	Remove deposited amyloid via monoclonal antibodies that bind specifically to misfolded or aggregated TTR; antibody-amyloid complexes are recognized and cleared by phagocytic immune cells [36, 37]
Gene editing	NTLA-2001, nexiguran ziclumeran	Permanent TTR gene inactivation using CRISPR-Cas9 in vivo gene editing; single-dose administration creates insertions or deletions in the chromosomal TTR gene, disrupting gene function and producing durable reductions in serum TTR [38, 39]

TTR: transthyretin; siRNA: small interfering RNA; mRNA: messenger RNA; TUDCA: tauroursodeoxycholic acid; CRISPR: clustered regularly interspaced short palindromic repeats.

Combination therapy pairing a stabilizer with a gene silencer represents an intellectually appealing strategy that addresses amyloidogenesis through complementary mechanisms: stabilizing circulating tetramers while simultaneously reducing their concentration. Preclinical rationale is strong, but clinical trial evidence is lacking. Ongoing studies are evaluating combination regimens, and results will inform whether dual therapy offers incremental benefit over monotherapy [40, 41].

Patient-specific factors may guide treatment selection even in the absence of comparative efficacy data. Gene silencing therapies require either intravenous infusions every 3 weeks (patisiran) or subcutaneous injections monthly to quarterly (eplontersen and vutrisiran), whereas oral stabilizers (tafamidis and acoramidis) offer the convenience of once- or twice-daily dosing without need for clinic visits. For patients with limited mobility or those living in geographically remote areas, oral therapy may be preferable. Conversely, patients with poor medication adherence might benefit from supervised administration.

Disease stage likely influences therapeutic response. All major trials excluded patients with NYHA class IV symptoms, reflecting the reality that advanced disease with severe functional limitation and high symptom burden may be less responsive to substrate reduction strategies. *Post hoc* analyses consistently demonstrate greater benefit when therapy is initiated earlier in the disease trajectory, before irreversible myocardial injury has occurred [41]. This observation underscores the critical importance of early diagnosis.

For ATTRv-CM, genotype may eventually inform treatment decisions. Variants associated with more aggressive phenotypes might warrant more intensive suppression strategies, whereas individuals with relatively stable disease might be adequately managed with stabilization alone. Biomarker-guided approaches using NT-proBNP trajectories, troponin trends, or imaging parameters could enable personalized treatment intensification or de-escalation, though prospective validation of such strategies is needed.

## Safety Considerations and Limitations

Despite remarkable progress, disease-modifying therapies for ATTR-CM are not without limitations and risks. Tafamidis and acoramidis have generally favorable safety profiles, though long-term consequences of chronic tetramer stabilization remain under investigation. Theoretical concerns about interfering with normal transthyretin physiologic functions, particularly thyroxine and retinol transport, have not manifested clinically in trials to date.

Gene silencing agents carry different safety considerations. Patisiran’s lipid nanoparticle formulation is associated with infusion-related reactions, typically mild and manageable with premedication, but rare severe reactions have been reported. Inotersen, though effective, is associated with potentially serious thrombocytopenia and glomerulonephritis, necessitating intensive monitoring that limits its use [42]. The newer ligand-conjugated siRNA (vutrisiran) and ASO (eplontersen) formulations appear to have improved safety profiles, though long-term data are still accumulating. Across the gene silencing class, reduced retinol transport warrants routine vitamin A supplementation with attention to ocular symptoms [21].

Near-complete suppression of transthyretin raises the question of whether the protein serves essential physiologic functions beyond thyroxine and retinol transport. Knockout mouse models lacking transthyretin develop normally and reproduce successfully, suggesting the protein is dispensable, at least in laboratory conditions. Human data from patients receiving gene silencing therapy for several years have not revealed unexpected toxicities attributable to transthyretin depletion, though vigilance is warranted.

CRISPR-based gene editing introduces unique considerations, including potential off-target chromosomal modifications, though next-generation sequencing analyses have not detected significant off-target editing in NTLA-2001 trials. The permanence of gene editing is both an advantage and a potential liability; irreversible modification precludes dose adjustment or treatment discontinuation should delayed toxicities emerge.

Cost represents a formidable barrier to access. At its 2020 United States list price of approximately $225,000 per year, tafamidis was estimated to have an incremental cost-effectiveness ratio of about $880,000 per quality-adjusted life-year (QALY) gained (95% uncertainty interval $697,000 to $1,564,000), far exceeding usual willingness-to-pay thresholds of $50,000 to $150,000 per QALY; at that price it was not cost-effective in any of 10,000 simulations at a $100,000 per QALY threshold, and a price reduction of roughly 93% would be required to meet it [43]. Treating all eligible United States patients at the list price was projected to add tens of billions of dollars to annual healthcare spending. Gene silencing therapies carry similarly high costs. The economic sustainability of novel therapeutics for chronic diseases affecting predominantly elderly populations is a pressing societal challenge. Value-based pricing models, outcomes-based reimbursement contracts, and international price negotiations will be essential to ensure equitable access.

Geographic disparities in diagnosis compound access challenges. ATTR-CM remains underdiagnosed in many regions due to limited awareness among clinicians, restricted access to advanced imaging modalities such as bone scintigraphy and cardiac magnetic resonance, and lack of specialized amyloidosis centers. Improving diagnostic pathways is a prerequisite for expanding treatment reach.

Several limitations of the current evidence base warrant emphasis. All pivotal trials excluded patients with NYHA class IV symptoms, so efficacy in advanced disease is unknown. No head-to-head trials compare stabilizers, gene silencers, or amyloid-depleting agents, and cross-trial comparison is confounded by differing endpoints and populations. Follow-up in the registration trials was relatively short (about 30 months) relative to the chronic course of ATTR-CM, leaving long-term efficacy and safety incompletely defined. Several trials used hierarchical or win-ratio composite endpoints that, although statistically efficient, do not map directly onto absolute event reductions and can be difficult to translate into individual patient prognosis. Many supporting analyses rely on surrogate measures such as NT-proBNP, troponin, and imaging parameters rather than hard clinical outcomes. Trial populations have under-represented women and patients of diverse genetic ancestry, including carriers of the V122I variant common in individuals of African ancestry, which limits generalizability. Finally, the efficacy and safety of combination regimens pairing stabilizers with gene silencers or amyloid-depleting antibodies remain unproven, and treatment-sequencing strategies are undefined.

## Future Directions

The next decade of ATTR-CM therapeutics will likely focus on several key priorities. Combination therapy trials evaluating stabilizers plus gene silencers, and potentially adding amyloid-depleting antibodies, will determine whether multi-pronged approaches offer superior outcomes to monotherapy. Early data from registries and compassionate use programs suggest that combinations are safe and well tolerated, but definitive efficacy data are needed.

Biomarker-guided treatment selection and monitoring represent another frontier. NT-proBNP, high-sensitivity troponins, extracellular volume fraction on cardiac MRI, and reticuloendothelial system uptake scores on bone scintigraphy all correlate with disease burden and prognosis. Integrating these markers into treatment algorithms could enable precision medicine approaches, identifying which patients require aggressive multi-drug regimens versus those who can be managed with single-agent therapy [44].

For ATTRv-CM, genotype-specific treatment strategies are emerging. Variants such as V122I, common in individuals of African ancestry, and T60A, prevalent in certain European populations, exhibit distinct natural histories and may respond differently to therapeutic interventions. Pharmacogenomic studies correlating transthyretin genotype with treatment response will refine personalized approaches.

Amyloid removal strategies beyond antibodies are in early development, including small molecules designed to disrupt fibril structure and promote clearance. If these agents prove effective, the vision of not merely halting disease progression but actually reversing organ damage may become reality.

The role of adjunctive therapies requires clarification. Heart failure medications including diuretics, beta-blockers, and renin-angiotensin-aldosterone system inhibitors have historically formed the backbone of ATTR-CM management, yet their efficacy in amyloid cardiomyopathy is poorly studied, and some agents may be poorly tolerated or even deleterious. Randomized trials evaluating conventional heart failure drugs specifically in ATTR-CM populations would address these knowledge gaps.

Finally, earlier diagnosis through population screening of at-risk cohorts, such as elderly men with unexplained left ventricular hypertrophy or heart failure with preserved ejection fraction, could shift the treatment paradigm toward prevention. Identifying ATTR-CM at asymptomatic or minimally symptomatic stages, when disease-modifying therapy is likely most effective, remains an aspirational goal with potential to dramatically improve long-term outcomes.

## Conclusions

ATTR-CM has transitioned from an untreatable and often unrecognized disease to a condition with multiple effective pharmacologic interventions. Tafamidis and acoramidis have established the clinical validity of transthyretin tetramer stabilization, demonstrating reductions in mortality and preservation of functional capacity and quality of life. Gene silencing approaches using siRNA and ASOs have achieved robust suppression of transthyretin production with emerging evidence of clinical benefit and potential disease modification. Novel strategies involving amyloid-depleting monoclonal antibodies and single-dose CRISPR gene editing hold promise for removing existing deposits and achieving permanent disease control. Despite these advances, challenges remain. The high cost of therapies threatens equitable access, particularly in resource-limited settings. Optimal patient selection, treatment sequencing, and combination strategies require further investigation. Long-term safety of profound transthyretin suppression and permanent gene editing must be carefully monitored. Most fundamentally, the majority of ATTR-CM patients remain undiagnosed, unable to benefit from effective therapies because their disease is not recognized. The convergence of diagnostic innovation, mechanistic understanding, and therapeutic innovation has expanded treatment options in ATTR-CM management. As the field advances, the focus must expand beyond efficacy in clinical trials to effectiveness in real-world populations, addressing disparities in diagnosis and access while continuing to push the boundaries of what is therapeutically possible. For patients with this once-fatal disease, effective disease-modifying therapy is now a reality, and the central challenge is to deliver it earlier and more equitably.

## Data Availability

This is a narrative review article. No new data were collected or generated. All data discussed in this manuscript are derived from previously published studies and publicly available literature cited herein.

## Abbreviations

ATTR-CM: transthyretin amyloid cardiomyopathy
ATTRwt-CM: wild-type transthyretin amyloid cardiomyopathy
ATTRv-CM: hereditary (variant) transthyretin amyloid cardiomyopathy
TTR: transthyretin
siRNA: small interfering RNA
ASO: antisense oligonucleotide
mRNA: messenger RNA
CRISPR: clustered regularly interspaced short palindromic repeats
NT-proBNP: N-terminal pro-B-type natriuretic peptide
NYHA: New York Heart Association
HR: hazard ratio
CI: confidence interval
RR: relative risk
RCT: randomized controlled trial
6MWD: 6-min walk distance
GLS: global longitudinal strain
LVEF: left ventricular ejection fraction
LVH: left ventricular hypertrophy
HFpEF: heart failure with preserved ejection fraction
CMR: cardiac magnetic resonance
KCCQ: Kansas City Cardiomyopathy Questionnaire
TUDCA: tauroursodeoxycholic acid
IV: intravenous
SC: subcutaneous
V122I: valine-to-isoleucine substitution at position 122
T119M: threonine-to-methionine substitution at position 119
